# From short-term engagements to meaningful and equitable global health partnerships

**DOI:** 10.1371/journal.pgph.0004011

**Published:** 2024-12-12

**Authors:** Shailendra Prasad, Eunice Kamaara, Bruce Compton

**Affiliations:** 1 Center for Global Health and Social Responibility, University of Minnesota, Minneapolis, Minnesota, United States of America; 2 Department of Philosophy & Religious Studies, Moi University, Eldoret, Kenya; 3 Catholic Health Association, Washington, DC, United States of America; PLOS: Public Library of Science, UNITED STATES OF AMERICA; Emory University, UNITED STATES OF AMERICA

The struggle for global health equity is complex, often fueled by a desire for quick solutions. Though global health should be a broad, ‘big-tent’ endeavor, quite often it gets simplified to the immediacy of clinical medicine. Short-term global engagements in global health (STEGH), like medical missions and student trips, embody this impulse. These, like many other aspects of global health, have roots in the late 19^th^ century as a largely neocolonial pursuit of “international medicine” [1]. These may also be driven by availability of opportunities and push towards broader marketization of global experiences—not just by the need of the respondents, but also by the supply of these opportunities [2]. While these activities may come from altruistic intentions, that of ‘hoping to help’, they often create more problems than they solve [3].

STEGHs may inherently be hampered by lack of context, thereby neglecting the unique needs and cultural nuances of communities, leading to interventions that are irrelevant, disruptive, or even harmful [4]. Despite overwhelming evidence that clinical care contributes to ~10% of the health of a community, emphasis on a predominantly biomedical model of health and healing ignores socio-cultural and sprititual perspectives that can predispose a rejection of health services by communities [5]. Imagine delivering a week-long diabetes management workshop in a community struggling with malaria—the mismatch is clear. This mismatch in disease-based approaches, or when the socio-cultural factors are not considered, can lead to lasting mistrust of health care systems, as has been seen in the rejection of vaccination or emergent Ebola care [6,7].

The drivers for engaging in STEGHs are often determined in far away places rather by local communities [8]. The ‘need’ is made manifest through a ‘partnership’ with a local entity, such as an NGO or an academic partner—a setting for an unequal relationship between a donor and recipient. These partnerships create a revolving door of well-meaning strangers; building trust and rapport takes time—an investment these fleeting interactions fail to make. Sustainability is another casualty; these programs often lack long-term funding and end when the grant or funding has been spent. Short-lived programs lead to disrupted healthcare systems and dependency on external aid, fostering a "band-aid" approach to complex issues rather than sustained systemic change. For example, it may lead health sector actors to prioritize perverse incentives rather than develop much needed sturctures for universal health coverage [9]. This is not a critique of global health partnerships but rather a call for partnerships that are meaningful and ethical, forming the foundation of all global health initiatives.

It’s time to move beyond the illusion of progress and focus on building sustainable solutions alongside local communities. The Advocacy for Global Health Partnerships (AGHP), with its secretariat at the Uganda National Academy of Sciences, has been bringing together people from various sectors, including academia, health sector actors, international donor agencies, faith-based organizations, ministeries of health, to articulate the need and tenets of ethical partnerships in global health [10]. Under its auspices, the Brocher Declaration laid out six principles for ethical short-term engagements in global health (see Box 1) [11]. The declaration has been endorsed by over fifty organizations from around the world and an active learning community of practice is leaning on each other to operationalize the principles. The Brocher Declaration primarily addressed STEGHs; AGHP is currently engaging in expanding those core principles to all partnerships in global health.

**Box 1**

Principles of the Brocher Declaration that AGHP will be expanding on make it relevant to *all* partnerships in global health

Six Principles of the Brocher Declaration.

1. Mutual partnership with bidirectional input and learning
2. Empowered host country and community define needs and activities
3. Sustainable programs and capacity building
4. Compliance with applicable laws, ethical standards, and code of conduct
5. Humility, cultural sensitivity, and respect for all involved
6. Accountability for actions

The efforts of the AGHP, of building consensus, and of creating a community of practice, work to socialize and normalize ethical practice. This deliberate process goes beyond disseminating information; it fosters understanding and promotes adoption of ethical principles. Currently there is no enforcement mechanism that ensures these ethical principles are followed in global health activities. AGHP has been encouraging the creation supportive environments for ongoing dialogue, observational learning, and critical reflection are important elements in this.

The unfortunate counterpoint to these efforts is the current structure of global health. Some actors in this space are too powerful, single disease approaches abound, and decisions about what will benefit communities are made in far-removed places [12]. There are ongoing attempts at making macro-level institutional changes, including the WHO, to improve global health, but we are still far away from realizing these at the micro-level [13].

Navigating the intricacies of international health systems, legal frameworks, and geopolitical dynamics is inherently complex, everchanging and therefore difficult. Yet, the potential to achieve significant, life-saving outcomes makes pursuing these partnerships essential to the future of global health. Threats to ethical, and hence equitable and effective, partnerships include funding and co-funding disparities between partners from high-income and low- income countries, unshared vision and priorities, skewed decision-making levels, and limited flexibility to minimize inequalities and make changes. Further, imbalances in power, privilege, position, income levels, and institutional resources create opportunities for the exploitation of partners, particularly those in low-income countries, which widens the disparities and limits the success and sustainability of partnerships [14].

So, what’s the path forward? The answer lies in fostering meaningful and effective partnerships with local communities as the driving force. The agenda needs to be set from the ground up fostering ownership of the work. Truly meaningful and equitable relationships demand a willingness to engage in open, honest dialogue that bridges diverse perspectives and expertise [14]. This involves actively seeking to understand the challenges and opportunities faced by different stakeholders, including local communities, healthcare workers, policymakers, and international partners. Power dynamics need to be assessed to ensure that any power difference is not manifested as a supremacy. The entities involved in the partnership should also identify their core values including principles of equity, solidarity and accountability. Too often global health partnerships are driven by convenience and do not aim for, or consider, lasting effects in communities. We need a commitment to practice these principles in recognition of the interrelatedness of all communities. Along with this there is a need for enforcement of these principles at the country level. We advocate that the Ministries of Health take a lead in enforcing this in individual countries. All parties involved in global health activities should evaluate their practices- from donors reviewing and monitoring their grant mechanisms to evaluating how their grantees are doing on these principles to participating NGOs and academic institutions reviewing their engagements and creating frameworks to assess compliance to these ethical principles. Consequences of not doing so imperils the health of all communities. As realities such as climate change has shown us, we either thrive together or perish together.

The essence of global health collaborations lies in the recognition that no single entity holds all the answers. Instead, it is through collective effort, mutual respect, and shared vision that impactful and sustainable health improvements can be realized. Rethinking global health partnerships with an equity lens will lead to more meaningful and productive collaborations. At the root of this is an acknowledgment that all the constitutents of earth- the humans, animals, plants and entities within- are one single community, a ‘single organism’ [15]. An absence of this framework makes us dichotomize between “us” and “them”, a non sustainable approach. Until we challenge this dichotomy and appreciate diversity we will continue to pay lip service to equity, shared vision, solidarity, mutual respect, among other values. This shift requires a long-term commitment to continuous dialogue, learning, and adaptation, grounded in the understanding that the strength of global health initiatives lies in our collective endeavor to foster a more resilient and responsive global health system.

## References

[1] Rowson M, Willott C, Hughes R, Maini A, Martin S, Miranda JJ, et al. Conceptualising global health: theoretical issues and their relevance for teaching. Globalization and Health. 2012 Dec;8:1–8. 23148788 10.1186/1744-8603-8-36 PMC3549856

[2] Bamberger A, Morris P, Yemini M. Neoliberalism, internationalisation and higher education: Connections, contradictions and alternatives. Discourse: Studies in the cultural politics of education. 2019 Mar 4;40(2):203–16.

[3] Lasker JN. Hoping to help: the promises and pitfalls of global health volunteering. In Hoping to Help 2016 Feb 19. Cornell University Press.

[4] Ventres WB, Wilson BK. Rethinking goals: transforming short-term global health experiences into engagements. Academic Medicine. 2020 Jan 1;95(1):32–6. doi: 10.1097/ACM.0000000000002841 31219810

[5] Bunker JP. The role of medical care in contributing to health improvements within societies. International journal of epidemiology. 2001 Dec 1;30(6):1260–3. doi: 10.1093/ije/30.6.1260 11821323

[6] Ghinai I, Willott C, Dadari I, Larson HJ. Listening to the rumours: what the northern Nigeria polio vaccine boycott can tell us ten years on. Global public health. 2013 Dec 1;8(10):1138–50. doi: 10.1080/17441692.2013.859720 24294986 PMC4098042

[7] Miller NP, Milsom P, Johnson G, Bedford J, Kapeu AS, Diallo AO, et al. Community health workers during the Ebola outbreak in Guinea, Liberia, and Sierra Leone. Journal of global health. 2018 Dec;8(2). doi: 10.7189/jogh.08.020601 30023054 PMC6030670

[8] Berry NS. Did we do good? NGOs, conflicts of interest and the evaluation of short-term medical missions in Sololá, Guatemala. Social Science & Medicine. 2014 Nov 1;120:344–51. 24834868 10.1016/j.socscimed.2014.05.006

[9] Advocacy for Global Health Partnerships. https://www.ghpartnerships.org/brocher, *accessed Aug 24*^*th*^, *2024*

[10] Prasad S, Aldrink M, Compton B, Lasker J, Donkor P, Weakliam D, et al. Global health partnerships and the brocher declaration: principles for ethical short-term engagements in global health. Annals of Global Health. 2022;88(1). doi: 10.5334/aogh.3577 35646612 PMC9122001

[11] Rumjaun A, Narod F. Social learning theory—albert bandura. Science education in theory and practice: An introductory guide to learning theory. 2020:85–99.

[12] Holst J. Global Health–emergence, hegemonic trends and biomedical reductionism. Globalization and health. 2020 May 6;16(1):42. doi: 10.1186/s12992-020-00573-4 32375801 PMC7201392

[13] Eccleston-Turner M, McArdle S. Accountability, international law, and the World Health Organization: A need for reform?. Global Health. 2017;11(1):27–39.

[14] Uganda National Academy of Sciences, 2024. Enacting an Ethic of Care and Responsibility in Global Health Partnerships: Kampala Uganda. https://unas.org.ug/wp-content/uploads/2023/04/Global-Health-Partnerships-27-October-CC-No-Pri_241027_104757.pdf *(accessed Nov 11*, *2024)*

[15] Plamondon KM, Brisbois B, Dubent L, Larson CP. Assessing how global health partnerships function: an equity-informed critical interpretive synthesis. Globalization and health. 2021 Dec;17:1–3. 34215301 10.1186/s12992-021-00726-z PMC8254362

